# A novel type 2 diabetes risk allele increases the promoter activity of the muscle-specific small ankyrin 1 gene

**DOI:** 10.1038/srep25105

**Published:** 2016-04-28

**Authors:** Rengna Yan, Shanshan Lai, Yang Yang, Hongfei Shi, Zhenming Cai, Vincenzo Sorrentino, Hong Du, Huimei Chen

**Affiliations:** 1 School of Medicine, Nanjing University, Nanjing, 210093, China; 2 Department of Endocrinology, Jinling Hospital Affiliated to Nanjing University School of Medicine, Nanjing, 210002, China; 3 Department of Endocrinology, Nanjing First Hospital Affiliated to Nanjing Medical University, Nanjing, 210006, China; 4 MOE Key Laboratory of Model Animals for Disease Study, Model Animal Research Center and the School of Medicine, Nanjing University, National Resource Center for Mutant Mice, Nanjing 210093, China; 5 Jiangsu Key Laboratory of Molecular Medicine, Nanjing, 210002, China; 6 Department of Urology, Drum Tower Hospital Affiliated to Nanjing University School of Medicine, Nanjing, 210008, China; 7 Department of Orthopedics, Drum Tower Hospital Affiliated to Nanjing University School of Medicine, Nanjing, 210008, China; 8 Molecular Medicine Section, Department of Molecular and Developmental Medicine, University of Siena, Siena, 53100, Italy

## Abstract

Genome-wide association studies have identified Ankyrin-1 (*ANK1*) as a common type 2 diabetes (T2D) susceptibility locus. However, the underlying causal variants and functional mechanisms remain unknown. We screened for 8 tag single nucleotide polymorphisms (SNPs) in *ANK1* between 2 case-control studies. Genotype analysis revealed significant associations of 3 SNPs, rs508419 (first identified here), rs515071, and rs516946 with T2D (P < 0.001). These SNPs were in linkage disequilibrium (r^2^ > 0.80); subsequent analysis indicated that the CCC haplotype associated with increased T2D susceptibility (OR 1.447, P < 0.001). Further mapping showed that rs508419 resides in the muscle-specific *ANK1* gene promoter. Allele-specific mRNA and protein level measurements confirmed association of the C allele with increased small ANK1 (sAnk1) expression in human skeletal muscle (P = 0.018 and P < 0.001, respectively). Luciferase assays showed increased rs508419-C allele transcriptional activity in murine skeletal muscle C2C12 myoblasts, and electrophoretic mobility-shift assays demonstrated altered rs508419 DNA-protein complex formation. Glucose uptake was decreased with excess sAnk1 expression upon insulin stimulation. Thus, the *ANK1* rs508419-C T2D-risk allele alters DNA-protein complex binding leading to increased promoter activity and sAnk1 expression; thus, increased sAnk1 expression in skeletal muscle might contribute to T2D susceptibility.

Type 2 diabetes (T2D) is a chronic metabolic disease with multifactorial pathogenesis, and genetic contribution to this disease is well recognized^1^,^2^,^3^. Recent genome wide association studies (GWAS) have identified over 70 new common single nucleotide polymorphisms (SNPs) associated with T2D, accounting for approximately 5.7% of the variance in T2D susceptibility. Data from additional case-control studies have substantiated the association of some SNPs with T2D and have suggested possible underlying physiological bases for T2D using more elaborate glucose metabolic measures. However, the majority of these SNPs are located near or within the introns of candidate risk genes; thus, it is difficult to interpret the biologic function of these susceptibility SNPs. Yet, a better understanding the functional significance associated with these SNPs would likely have important therapeutic implications.

Ankyrin 1 (ANK1) was first identified as a functional link between integral membrane proteins and the underlying spectrin network in erythroid cells^4^. In humans, mutations in *ANK1* cause hereditary spherocytosis; therefore, ANK1 has been considered pivotal in stabilizing the membrane structure of erythrocytes^5^. In striated muscles, muscle-specific small ANK1 (sAnk1) isoforms, encoded by shorter transcripts driven by the activity of a second internal *ANK1* promoter^6^,^7^, have been implicated in stabilizing the sarcoplasmic reticulum around the myofibrils^8^,^9^,^10^,^11^,^12^. The *ANK1* variants rs6474359 and rs4737009 were formerly considered to be linked with diabetes, when HbA1c was considered to be a primary maker for this disease^13^. These variants might influence erythrocyte lifespan and lower HbA1c levels without affecting plasma-glucose levels. Recently, the analysis of GWAS data showed a significant association between T2D and SNPs in *ANK1*, rs515071, and rs516946, and the importance of extended analyses was confirmed in multi-ethnic groups^14^,^15^. The association of these variations with T2D was independent of associations related to the HbA1c levels. However, the contribution of these *ANK1-*locus SNPs to T2D susceptibility remains unclear. As a further complicating factor, *ANK1* produces several alternatively spliced transcripts, some of which lack large segments that include whole functional domains^16^. The diversity of the ankyrins suggests that, in addition to their well-known role in the membrane skeleton, ANK1 protein isoforms might serve other more specific roles in different cell types.

SNPs rs515071 and rs516946 and SNPs rs6474359 and rs4737009 are in perfect linkage disequilibrium (LD) with each other, respectively, in Europeans; however, they are all located in *ANK1* intronic regions. We hypothesized that functional variant(s) should be in high LD with these reported SNPs. Searching for regulatory SNPs within the LD region might be a good strategy for revealing the underlying molecular genetic mechanism(s) contributing to the associations identified by GWAS. In this study, we identified candidate T2D risk SNPs in the *ANK1* locus and examined their functional effects on the expression of ANK1 and its isoforms in skeletal muscles, as well as on glucose uptake *in vitro*. We suggest that the results of these analyses might explain the previous GWAS-based identification of T2D-associated variants in this region.

## Results

To identity the association of SNPs with T2D in Chinese patients, we performed case-control studies with 2-stage analysis of 1174 patients with diabetic mellitus. The clinical characteristics and demographics of both stages are summarized in Table 1. Analysis in stage 1 showed that rs508419 (P = 4.830 × 10^−4^), rs515071 (P = 3.630 × 10^−4^), and rs516946 (P = 1.410 × 10^−4^) of *ANK1* were significantly associated with T2D (Table 2). Furthermore, these 3 SNPs showed nominally significant association with levels of HbA1c measured among the diabetic population, even after adjustments for glucose levels, sex, and age (all P < 0.05). The other 5 originally selected candidate SNPs were excluded because they were not associated with T2D or HbA1c levels (P ≥ 0.05); however, rs6474359 was weakly associated with Hb levels when adjusted for sex and age. Rs516946, rs515071, and rs6474359 in *ANK1* were previously associated with diabetes and HbA1c^1^,^13^,^14^,^15^. In our stage 1 study, we further identified SNP rs508419 as a novel susceptibility variant for T2D.

**Figure 1:**
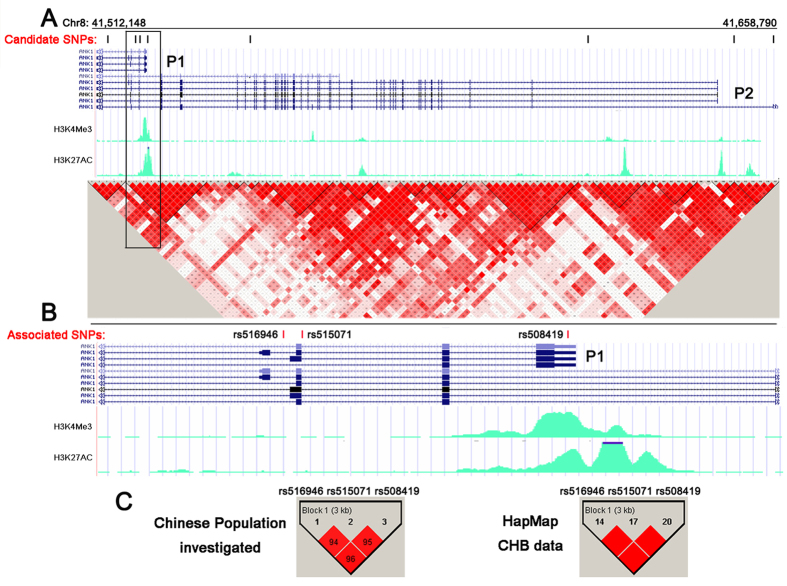
Structure of the *ANK1* risk locus and locations of the investigated SNPs. (**A**) Distribution of 8 candidate SNPs within the different transcripts of *ANK1*. The UCSC Genome Browser diagram shows that the *ANK1* gene is transcribed from right to left, with P1 and P2 promoter-specific transcripts. The green peaks are related to the open chromatin in HSMM cells. Haplotype analysis results from the HapMap CHB genotype data are shown in a colour-coded scale, expressing the logarithm of odds (LOD) value for the LD (red: LOD score ≥ 2 and D′ = 1; pink: LOD score ≥ 2 and D′ < 1; blue: LOD score < 2 and D′ = 1; white: LOD score < 2 and D′ < 1). (**B**) T2D risk SNPs located in the sAnk1 transcript (magnified views from the boxed area in Fig. 1A). The results from the 2-stage case-control studies confirmed that rs508419 was localized to the *ANK1* P1 promoter and that 2 additional variants (rs516946 and rs515071) were associated with T2D. (**C**) LD mapping of the 3 associated SNPs (P < 0.01; left) examined in this study and the HapMap Phase II CHB genotype data (P < 0.01; right). The LD structures of all SNPs (including the 3 risk SNPs) were analysed using Haploview 4.2.

Further stage 2 analysis confirmed that rs508419 (odds ratio [OR] 1.531; P = 2.850 × 10^−4^), as well as rs515071 (OR 1.468; P = 1.860 × 10^−4^) and rs516946 (OR 1.495; P = 5.880 × 10^−4^) were associated with an increased risk for T2D (Table 3). The Haploview 4.2 program (www.broad.mit.edu_mpg_haploview) showed the evaluated LD structure of these SNPs (Fig. 1A). In particular, rs508419, rs515071, and rs516946 were in strong LD in the investigated population and within the HapMap Chinese Han Beijing population (CHB) genotype data (all *r*^*2*^ > 0.8; Fig. 1C). The haplotype of the 3 risk alleles (C at rs508419, C at rs515071, and C at rs516946) further confirmed the association with T2D risk: OR =1.477 (95% confidence interval [CI]: 1.203–1.814, P = 1.870 × 10^−4^; Table 3).

To localize the 3 SNPs with regards to the alternatively spliced transcripts, we determined that they were all localized within introns of long *ANK1* isoform transcripts, whereas rs508419 was located at the alternative promoter 1 (P1) that is responsible for the muscle-specific expression of sAnk1 isoforms (Fig. 1B). This region is characterized by an open and accessible chromatin configuration in muscle (Fig. 1A,B), as it overlaps the Encyclopedia of DNA Elements (ENCODE)-based promoter and regulator elements binding sites that are marked by active histone H3 lysine modifications (H3K4me3, and H3K27ac) in human skeletal muscle cells and myoblasts (HSMMs)^17^,^18^.

**Figure 2:**
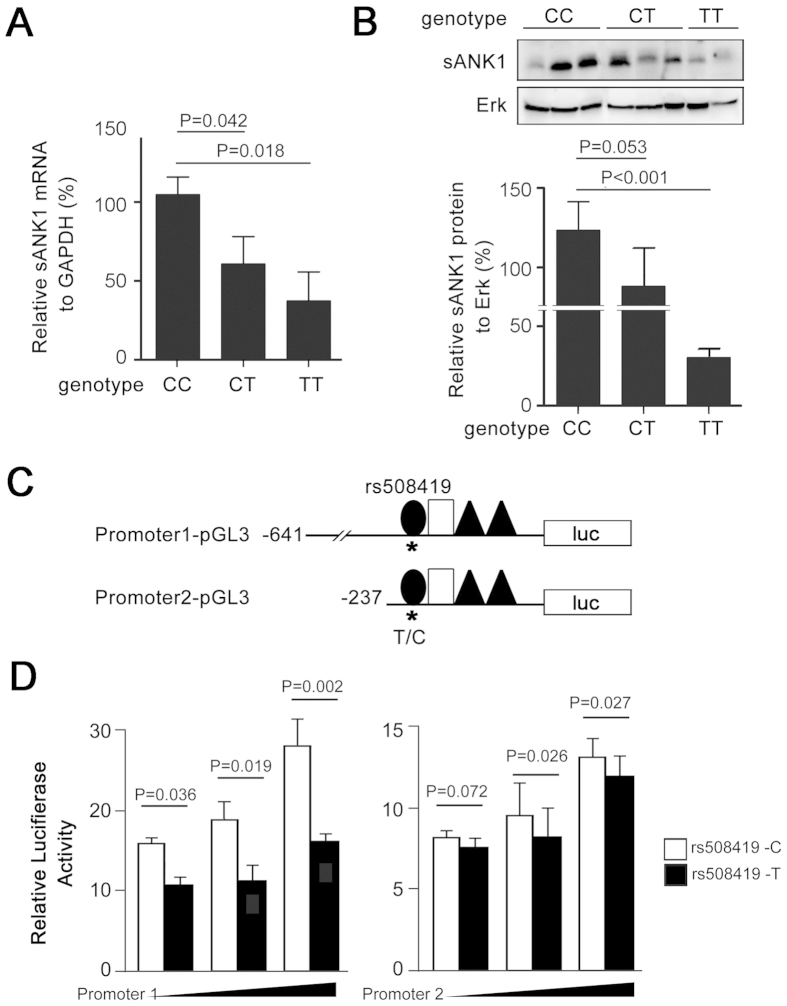
Dissection of the effect of SNP rs508419 on the transcriptional activity and expression of sAnk1. **(A**,**B**) Expression of sAnk1 mRNA (**A**) and protein (**B**) increased in human skeletal muscle tissues with the rs508419 C allele, compared to that observed in samples carrying the T allele. The results shown represent the mean ± S.D. Differences among the CC, CT, and TT groups were compared using one-way ANOVA testing, followed by an LSD test. Twenty-six muscle samples were analyzed including 17, 5, or 4 samples from subjects with the CC, CT, or TT genotype, respectively (Supplementary Table S3). Protein samples were available from 7 subjects with the CC genotype. (**C**) The *ANK1* gene P1 promoter corresponding to positions −641 to +82 or −237 to +82 relative to the transcription initiation site (+1) with allele C or T at site c. − 199 (rs508419) were cloned in the pGL3 plasmid, resulting in the construction of 4 plasmids: Promoter1-pGL3-C/T and Promoter2-pGL3-C/T. (**D**) The activity of the *ANK1* P1 and P2 plasmid types and the empty pGL3-Basic vector were studied in C2C12 cell transfectants. The results shown represent the mean ± S.D. of at least 3 independent transfection experiments. *P* values were calculated by Student’s *t*-test.

Because sAnk1 is highly expressed in skeletal muscle, we evaluated its mRNA-expression and protein-production levels in primary skeletal muscle from subjects with rs508419 C/C, C/T, and T/T genotypes. Compared with the C/T or T/T genotype, the relative expression of sAnk1 mRNA significantly increased in samples carrying the C/C genotype (Fig. 2A). Setting the mRNA expression level in subjects with the C/C genotype as “100%,” the relative expression levels of sAnk1 in individuals with C/T and T/T genotypes were 57% and 36% (P = 0.042 and P = 0.018), respectively. Subjects carrying the C/C genotype further showed higher sAnk1 protein levels than those with the T/T genotype (Fig. 2B; P < 0.001).

To interrogate allelic differences in the transcriptional activity of rs508419, we cloned DNA sequences containing either the T or C allele into a promoterless luciferase vector. The resulting Promoter1-pGL3 construct contained c. − 641 to c. + 82 and Promoter2-pGL3 contained c. − 237 to c. + 82, where the transcriptional start site of sAnk1 was considered as “+1” (Fig. 2C). As shown in Fig. 2D, the C allele in both plasmids demonstrated significantly higher luciferase activities in a dose-dependent manner, compared to those obtained using T allele constructs, with a 70% (P = 0.002) increase at the highest dose (Fig. 2D).

**Figure 3:**
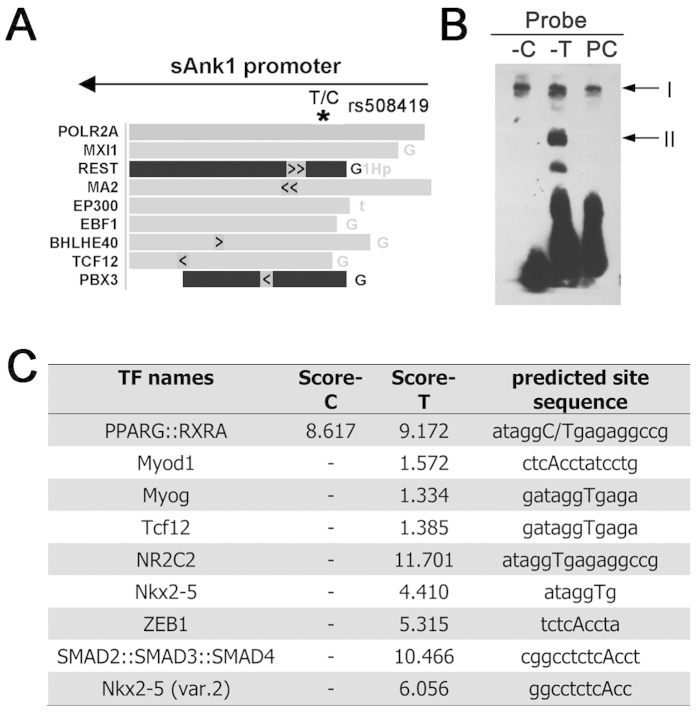
The T2D C risk allele of rs508419 alters transcription factor binding. **(A**) The genomic rs508419 SNP site is localised to a transcription factor-binding region, as determined by analysing ChIP-seq data from the ENCODE project. The arrow indicates the direction of sAnk1 transcription. (**B**) EMSA analysis with C2C12 cell extracts and oligonucleotide probes containing the C and T alleles of the *ANK1* P1 promoter. PC, positive control. (**C**) The different binding affinities of transcription factors observed with 2 EMSA oligonucleotide probes obtained from the JASPAR database. A high score is predicted to be associated with strong binding of proteins to specific DNA sequences.

To further analyse the region around rs508419, we utilised the ENCODE data in the UCSC Genome Browser. The ENCODE project comprises 1,640 data sets, from 147 different cell types^17^. According to this analysis, the rs508419 region could bind several transcriptional factors, as shown in Fig. 3A.

Accordingly, we performed electrophoretic mobility shift assays (EMSAs) using nuclear protein lysates extracted from murine skeletal muscle C2C12 myoblast cells to evaluate differences in transcription factor binding to either allele of rs508419. Multiple DNA-protein complexes were observed in the EMSAs (Fig. 3B). Complex I was evident for both alleles. At least 1 protein complex (II) was observed to bind specifically to the non-risk (T) allele of rs508419. When probe-T competitor was added, DNA-protein complex II nearly disappeared (Supplementary Fig. S1). Preferential protein binding to the T allele was also detected in 3T3-L1 cells for a complex with similar mobility (Supplementary Fig. S2).

We further computationally analyzed the potentially bound protein(s) using the online JASPAR database, where a high score is predicted to be associated with strong binding of proteins to specific DNA sequences. As showed in Fig. 3C, several transcription factors were predicted to specifically bind to the T allele, but not to the C allele, including Myod1, Myog, Tcf12, NR2C2, Nkx2-5, ZEB1, and SMAD2::SMAD3::SMAD4. Although the specific binding factors in our study remain undetermined, our analysis shows that band II potentially contains candidate proteins that exhibit T allele-specific binding to rs508419, which might contribute to promoter activity.

**Figure 4:**
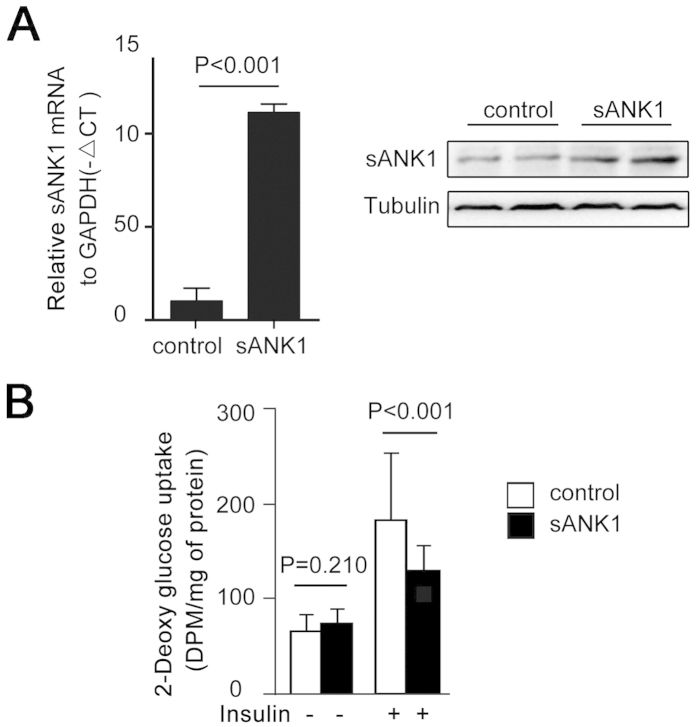
Overexpression of sAnk1 decreased glucose uptake in C2C12 cells. (**A**) The efficiency of pEGFP-c1-sAnk1 transferred in C2C12 cells. C2C12 cells were transfected with the pEGFP-c1-sAnk1 plasmid. After differentiation, total RNA and protein isolates were analyzed by qPCR and western blotting, respectively. (**B**) Glucose uptake in C2C12 cells after transfection with the pEGFP-c1-sAnk1 plasmid and subsequent differentiation. The data shown are the mean ± S.D. *P* values were calculated by Student’s t-test.

To discover whether sAnk1 levels affect the insulin resistance of skeletal muscle, we cloned the coding sequence of sAnk1 into the plasmid pEGFP-c1. The mRNA and protein levels of sAnk1 in C2C12 cells significantly increased after transfection with pEGFP-c1-sAnk1 (Fig. 4A). Glucose uptake was measured in C2C12 myotubes in basal (without insulin) and stimulated (100 nM insulin, 30 min) states. As shown in Fig. 4B, insulin stimulation caused a significant decrease in 2-DOG uptake in C2C12 cells, concomitant with excess sAnk1 expression.

## Discussion

The *ANK1* gene, located on chromosome 8p11.1, is a member of the ankyrin gene family that encodes proteins that participate in the organization of membrane domains and proteins in connection with the cytoskeleton. There are 8 main different splice variants that are often divided into 2 types, namely the long and short isoforms. The long isoforms, named as ANK1, was first found in erythrocytes and acts as a linker between integral membrane proteins and the underlying spectrin-actin cytoskeleton. Additional ANK1 isoforms have also been found in most other cells and tissues^4^. In striated muscles, sAnk1 isoforms are highly expressed, helping stabilize the sarcoplasmic reticulum in relation to the contractile apparatus^8^,^12^,^19^. Different isoforms of sAnk1 are driven by the same promoter (named as P1 in this study), independently of the P2 promoter, which is not active in muscle cells. More recently, GWAS in multi-ethnic groups have identified SNPs in *ANK1*, in particular rs515071 and rs516946, as susceptibility variants for T2D. These sites likely represent a common locus for T2D across multiple ethnic populations.

In this study, we screened *ANK1* SNPs and identified a plausible functional regulatory variant, namely rs508419: C > T. We also demonstrated its allelic effects on sAnk1 mRNA and protein expression in skeletal muscle. We provided evidence suggesting that the C allele of rs508419 disrupts the binding of transcriptional regulators and thereby increases transcriptional activity at the *sANK1* promoter, leading to higher expression in muscle. Furthermore, we tested the muscle small ankyrin1 isoform 5 (sAnk1.5) as a repetitive variant of sAnk1^20^ and showed that sAnk1 overexpression resulted in decreased glucose uptake in C2C12 myotubes. These findings implied that increased sAnk1 expression in skeletal muscle might represent a molecular genetic contributor to T2D susceptibility.

Rs515071 and rs516946, identified by different GWASs of T2D, are in perfect LD with each other and are located within introns 1 and 2 of the sAnk1 transcript, respectively. We determined that regulatory variant(s) at this locus were in high LD with rs515071 and rs516946, which pinpointed the underlying reason for the association of *ANK1* with T2D risk. Stage 1 analysis identified the risk association of rs508419-C, rs515071-C, and rs516946-C alleles with T2D in Chinese patients. A stage-2 study further confirmed these associations in an enlarged population. The prevalence of the CCC haplotype, comprising the 3 SNP risk alleles, provided solid support for this relationship in the patients studied. We therefore identified rs508419 as a novel susceptibility variant for T2D at the *ANK1* locus.

Specific mapping showed that rs508419, rs515071, and rs516946 are all localized within introns of *ANK1*. However, the localization of the 3 SNPs differed with respect to the sAnk1 transcript. Rs508419 localizes to the promoter of the sAnk1 transcript, whereas rs515071 and rs516946 are located within introns of this isoform. In addition, rs508419 is in high LD with rs515071 and rs516946. These results suggested that rs508149 might be a functional SNP and thus might contribute to the molecular function of the *ANK1* locus identified through the GWAS. We further demonstrated that the rs508419-C allele is associated with increased mRNA and protein expression of sAnk1 in primary human skeletal muscle. In agreement, a luciferase reporter assay showed that the rs508419-C allele was associated with transcriptional upregulation at the sAnk1 promoter. Together, these findings implicated increased expression of the sAnk1 isoform, containing rs508419, in T2D susceptibility. We note, however, that the primary skeletal muscle expression data were drawn from a small number of samples, and that genotypic effects in expression studies of quantitative trait loci are often complex with confounding effects, such as inter-individual environmental exposures.

Transcriptional activity is usually regulated by DNA-protein binding at promoter regions. Rs508419 is located at the P1 promoter of sAnk1, overlapping the ENCODE-annotated transcription factor binding sites within a region of open and accessible chromatin. EMSA analysis indicated that the C allele of rs508419 altered the binding of transcriptional regulators at the sAnk1 promoter. The JASPAR database further predicted several candidate transcription factors that exhibit differential binding to rs508419-C or T alleles, some of which have been reported to play a critical role in regulating energy and lipid homeostasis^21^ or skeletal muscle differentiation^22^,^23^. We have identified several potential transcription factors that bind to rs508419, but the exact transcript factors with different binding affinities to the C or T alleles, as well as the underlying molecular mechanism, requires further investigation.

To date, few common T2D-susceptibility variants have been shown to associate with insulin resistance. In this study, we demonstrated that excess sAnk1 expression led to decreased glucose uptake in C2C12 cells. Because the rs508419C allele upregulated sAnk1 transcription and protein expression in skeletal muscle, it is plausible that rs508419C promotes the development of diabetes through insulin resistance.

In addition, rs508419 was associated with T2D with almost equal significance as was rs516946 (rs508419: P = 2.4 × 10^−5^, n = 63,390; rs516946: P = 7.3 × 10^−7^, n = 69,033) in the Stage-1 data from the DIAGRAM publication, in which the rs508419 locus was first linked to T2D^1^. Of the trio of associated SNPs in LD identified in this study, only rs516946 was available on the Metabochip used in the DIAGRAM study. The rs516946 SNP was further examined in the Stage-2 study and identified as the lead SNP at this locus. Clinical analysis showed an association between rs516946 and decreased insulinogenic and disposition indexes, suggesting a possible role for T2D variants near Ank1 in beta-cell dysfunction^15^. Imamura *et al.*^14^ showed that the levels of either sAnk1 or ANK1 were similar in islets between subjects with and without diabetes; but only four individuals were detected. Palmer *et al.*^24^ selected rs508419 as a proxy SNP of the previously reported T2D-risk gene *ANK1* to evaluate its role in T2D-related quantitative traits in GUARDIAN Hispanic Americans, but no significant association was observed. The present study showed a low OR of rs508419 for diabetes. The following considerations may explain this inconsistency: 1) racial differences between these populations were investigated; 2) many variants with modest associations are often ignored after multiple testing correction in GWASs^25^; and 3) regulation of sAnk1 in the long forms and in other tissues partly added to such association, since both long and short isoforms of sAnk1 are expressed across a range of tissues. However, the data presented in the present study cannot address these possibilities. Transgenic and conditional knock out animal models should be constructed to help us understand pathogeneitc of SNPs and (s)ANK1 in diabetes.

The muscle-enriched sAnk1 isoform is anchored to the sarcoplasmic reticulum and is localized to the Z and M lines of internal myofibrils^5^,^10^,^11^,^26^, contributing to the maintenance of skeletal muscle structure and function^12^. The expression of sAnk1 in muscle positively associated with SERCA and GLUT4 expression (Supplementary Fig S4). However, the expression of SERCA and GLUT4 in insulin resistance was paradoxically reported^27^,^28^,^29^,^30^. Funai, K. *et al.*^27^ showed SERCA downregulation will increase insulin sensitivity via Ca^2+^ homeostasis, while Safwat, Y. *et al.*^28^ suggested that upragulated SERCA was associated with insulin resistance. Increased expression of GLUT4 in skeletal muscle could increase insulin sensitivity^29^, while the increased expression of GLUT4 was also observed with insulin resistance, when insulin is unable to recruit GLUT4 to the cell surface^30^. Ackermann, M. A. *et al.*^5^ and our group^12^ have shown that sAnk1 knock-down can directly lead to SERCA downregulation. We, therefore, proposed that upregulation of SERCA resulted from increased sAnk1 might contribute to altered Ca^2+^ homeostasis and then insulin resistance, and GLUT4 was consequently accumulated in muscle. However, the mechanism by which sAnk1 in skeletal muscle might be involved in T2D remains uncertain.

Our data do not preclude the existence of additional functional variants, and/or additional/alternative mechanisms associated with this locus. In this study, we screened tagging SNPs located at regulatory regions, with minor allele frequencies (MAFs) of >0.1, and several reported variants. An additional 10 SNPs that are linked with rs508419, rs515071, and rs516946 were also investigated. Rs6989203 is located at nucleotide position −953 of sAnk1, whereas other SNPs are located far from the regulatory element, or within the introns. Similar with rs508419, the DIAGRAM study also showed that rs6989203 is related with the risk for T2D before multiple analysis^24^. Because rs6989203 is located at the regulatory region for transcription^7^, we could not exclude its effect on altered transcription, which contributed to T2D. However, it is difficult to rule out this SNP and other variants located at exons or introns with MAFs <0.1, which might also affect *(s)ANK1* functions.

Although we could not identify the exact transcript factor involved, DNA-protein binding may mediate different transcription activities related to rs508419. Several potential transcript factors binding rs508419 have been found and under investigation in our lab. Furthermore, epigenetic regulation might occur as an alternative mechanism for modulating *ANK1* expression in T2D, considering that recently, 2 independent epigenome-wide association studies of Alzheimer’s disease cohorts identified methylation signals in *ANK1* associated with Alzheimer’s disease^31^,^32^,^33^.

In summary, this work revealed a novel T2D-risk SNP, provided a biological explanation for existing T2D GWAS findings, and paves the way for future research on the role of sAnk1 as a diabetes candidate gene in skeletal muscle. Because GWASs have identified an abundance of risk loci for T2D, elucidating mechanisms to explain how variants influence the expression or function of target genes will facilitate the discovery of biological gene candidates and novel pathways involved in disease pathology.

## Methods

### Subjects

We performed 2 stages of case-control studies, included Chinese subjects with or without diabetes (range 21–90 years of age). The stage-1 cohort consisted of 441 patients (mean age 59.3 ± 11.1 years, range 40–79 years) and 411 age- and sex-matched healthy controls. The stage-2 cohort included 733 patients (mean age 57.1 ± 13.1 years, range 21–90 years) and 733 age- and sex-matched healthy controls. T2D was diagnosed according to the World Health Organization recommendations^34^. Detailed interviews and regular laboratory analyses were performed. Age- and sex-matched healthy individuals entering medical centres for routine medical exams were enrolled from same area at similar periods between 2009 and 2010, as control subjects. Those who showed an abnormal fasting blood glucose level (≥6.1 mM) or had other metabolic diseases were excluded. In addition, patients with cancer or acute or chronic diseases were excluded. Another 26 volunteers were enrolled from Nanjing Drum Tower Hospital, affiliated with the Medical School of Nanjing University, and skeletal muscle tissues were collected by biopsy. The study was approved by the Ethics Committee of the Medical School of Nanjing University and was conducted in accordance with all ethical guidelines. Written informed consent for an interview and a blood sample donation was obtained from each participant.

### Genotyping

All *ANK1* SNPs were retrieved from the HapMap database covering 144.4 kb (release 24/phase II Nov08, from the NCBI B36 assembly, dbSNP b126; CHB; MAF ≥ 0.10) and clustered with similar r^2^ values for one threshold (0.8) (Fig. 1A). The SNP clusters located in regulatory regions were selected for further study, as were the reported associated SNPs. Eventually, 8 SNPs (Fig. 1A) were selected for use in the present study. Detailed information for these SNPs is provided in Supplementary Table S1.

Genomic DNA, where applicable, was extracted from whole blood collected in EDTA from patients and control subjects using the Tiangen Blood DNA Kit, according to the manufacturer’s instructions (Tiangen, Beijing, China). Genomic DNA samples were genotyped for selected SNPs in sAnk1 using TaqMan Genotype assays (Applied Biosystems, Inc., Foster City, CA, USA), high-resolution melting (HRM), or polymerase chain reaction-restriction fragment length polymorphism (PCR-RFLP) analysis. The specific genotyping for either SNPs and the sequences of the PCR primers are shown in Supplementary Method and Table S2.

### sAnk1 expression analysis

Skeletal muscle tissue biopsies were collected and divided into 3 groups based on the rs508419 genotype: C/C (n = 17), C/T (n = 5), and T/T (n = 4). No significant differences in sex or age were found between the 3 genotype groups (Supplementary Table S3). Muscle tissues were obtained at biopsy and immediately frozen in liquid nitrogen and stored at −70 °C until further analysis.

*mRNA analyses.* Total RNA was extracted from the skeletal muscle samples using the TRIzol method (Takara), and cDNA synthesis was performed using the PrimeScript RT Reagent Kit (Takara). Real-time PCR was used to quantify mRNA expression levels of sAnk1 using primers that specifically amplify this isoform. The sequences of the primers used for quantitative PCR (qPCR) are shown in Supplementary Table S4. The forward primer used to evaluate the expression of sAnk1 transcript is located at the sequence named as 39a (Supplementary Fig. 3), which is comprised of part of intron 39 of *ANK1* and the first exon of *sANK1*. Relative fold-changes in cDNA expression detected by qPCR were calculated using the 2^−ΔΔCT^ method (AB7300 real-time PCR system, Applied Biosystems).

*Protein analyses.* Muscle specimens were homogenized using a TissueLyser II (QIAGEN, Venlo, The Netherlands) in NP40 lysis buffer and incubated end-over-end at 4 °C for 45 min. All homogenates were cleared by centrifugation (12,000 × *g*, 20 min, 4 °C), and the lysates were recovered and stored at −80 °C. Protein concentrations were determined using the BCA Protein Assay Kit (Thermo Scientific, Waltham, MA, USA). Equal amounts of protein were loaded and separated by SDS-PAGE. The membranes were incubated overnight with appropriate primary antibodies. Bound antibodies were then visualized using alkaline phosphatase-conjugated secondary antibodies. The abundance of sAnk1 was quantified using an antibody targeting the carboxy terminus of ANK1 (Catalogue no. ab58698, Abcam, Cambridge, UK). ERK1/2 and α-tubulin antibodies (Catalogue Nos 4695 and 2144, respectively) were acquired from Cell Signaling Technology (Danvers, MA, USA). Band intensities were evaluated using the US National Institutes of Health Image J 1.32j software.

### Cell culture

The murine muscle myoblast C2C12 cell line (American Type Culture Collection ATCC CRL-1772, Manassas, VA, USA) was used to study from expression and protein binding to the sAnk1 promoter. C2C12 cells were maintained in Dulbecco’s Modified Eagles’ Medium (DMEM) with 10% foetal bovine serum.

### Dual luciferase transcriptional reporter assays

The promoter region of sAnk1 including rs508419 was PCR amplified from genomic DNA and cloned in the pGL3 vector (Promega, Madison, WI, USA). Two sets of vectors were constructed with a long fragment from position c. − 641 to c. + 82 of the sAnk1 transcript (Promoter 1) and a short fragment from position c. − 237 to c. + 82 (Promoter 2). In total, 4 different plasmids were prepared (Fig. 2C), including Promoter 1-pGL3 with C at c. − 199 (Promoter 1C), Promoter 1-pGL3 with T at c. − 199 (Promoter 1T), Promoter 2-pGL3 with C at c. − 199 (Promoter 2C), and Promoter 2-pGL3 with T at c. − 199 (Promoter 2T) (Fig. 2C). The primer sequences used to generate these constructs are shown in Supplementary Table S5.

C2C12 cells were transfected with 1, 1.5, or 2 ug each plasmid or the empty pGL3-Basic vector (Promega), as well as 200 ng of the pRL-CMV plasmid (Promega) as an internal control to assess the transfection efficiency, using the Lipofectamine™ 2000 Transfection Reagent (Life Technologies Corp., Carlsbad, CA, USA). The resultant firefly luciferase activities were measured using GloMax 96 (Promega). Relative luciferase activities were calculated after normalising the luciferase activity of each experimental sample to that observed after transfection with the empty vector (set to 1.0).

### EMSAs

Two sets of complementary 22-mer oligonucleotides with biotin end labelling (Supplementary Table S4) were generated by Zoonbio Biotechnology Co., Ltd. (Nanjing, China), which were centred on rs508419 (T/C) with T or C alleles. We annealed each set to create double-stranded oligonucleotides; the sequences of which are specified in Supplementary Table S6. Nuclear protein lysates were extracted from C2C12 cell pellets with the NE-PER Nuclear Extraction Reagent Kit (Thermo Scientific), and protein concentrations were determined with the Pierce BCA Protein Assay Kit (Thermo Scientific). EMSAs were performed with the LightShift Chemiluminescent EMSA Kit (Thermo Scientific), according to the manufacturer’s instructions.

The JASPAR program (http://jaspar.genereg.net/) was used to computationally predict transcription factors that might differentially bind at rs508419 by contrasting the predictions and/or scores generated for each SNP allele^35^,^36^. Transcription factor ChIP-seq data for the region overlapping rs508419 were obtained from the ENCODE project^17^.

### Glucose uptake assay

#### Plasmid construction

The coding sequence of sAnk1 (GenBank: Accession No. U73972) was generated and cloned into pEGFP-C1 (GENEWIZ Biological Technology Co., Ltd., South Plainfield, NJ, USA). The pEGFP-c1-sAnk1 construct was verified through sequencing.

#### Transfection and differentiation

C2C12 cells were plated in 24-well plates and allowed to grow to 70% confluence. Transfections were performed with Lipofectamine 2000 (Invitrogen), according to the manufacturer’s protocol. Briefly, 1 ug pEGFP-c1-sAnk1 and 2 uL Lipofectamine 2000 were first diluted into 50 uL serum-free medium each and then mixed. The mixtures were then allowed to incubate for 20 min at room temperature and added dropwise to each culture well containing 400 uL serum-free medium. At 4 to 6 h post-transfection, the medium was exchanged with fresh complete medium. After the myoblasts achieved confluence, differentiation into myotubes was induced by incubation for 6–7 days in DMEM supplemented with 2% horse serum, which was changed every 2 days. Some cells were lysed for sAnk1 mRNA and protein determinations.

#### Glucose uptake measurement

Cells were washed in Krebs–Ringer phosphate (KRP) buffer and serum-starved in KRP for 2 h. Next, the cells were incubated in KRP with or without 100 nM insulin for 30 min. Then, they were incubated with 1 μCi [^3^H]2-deoxyglucose (PerkinElmer, Boston, MA, USA) and 0.1 mM 2-deoxyglucose for 60 min. After incubation, the cells were washed 3 times with ice-cold PBS and dissolved in 1 N NaOH. The solution was neutralized with 1 N HCl and the radiolabeled glucose in solution was assayed using a liquid scintillation counter.

### Statistical analysis

All statistical analyses were performed using the SPSS statistical program, version 16.0 (SPSS, Chicago, IL, USA). The data are shown as the mean ± SD or the percentage. A case-control association between the genotypes and T2D status was analysed by the Chi-square test, and ORs are shown with 95% CIs. The case-control study, pairwise LD estimation, and haplotype reconstructions were performed using the SHEsis software platform (http://analysis.bio-x.cn) and Haploview 4.2 software. One-way analysis of variance (ANOVA) was used to determine the statistical significance (2-tailed analysis) between 3 experimental groups. Multiple comparisons between groups were then assessed using the Tukey–Kramer post-hoc test (for parametric data). The Student’s *t*-test was used for continuous variables with 2 groups. Statistical significance was set at P < 0.05.

## Additional Information

**How to cite this article**: Yan, R. *et al.* A novel type 2 diabetes risk allele increases the promoter activity of the muscle-specific small ankyrin 1 gene. *Sci. Rep.* **6**, 25105; doi: 10.1038/srep25105 (2016).

## Supplementary Material

Supplementary Information

## Figures and Tables

**Table 1:** Baseline characteristics and demographics of the patients and controls enrolled in Stage 1 and Stage 2.

	Stage 1			Stage 2		
	Cases	Controls	P	Cases	Controls	P
Number	441	441		733	733	
Male, n (%)	225 (51.0%)	238 (54%)	NS	441 (60.2%)	454 (61.9%)	NS
Age, yr	59.3 ± 11.1	59.3 ± 11.2	NS	57.1 ± 13.1	56.9 ± 13.1	NS
Smoking, n (%)	74 (16.9%)	–	–	138 (19.1%)	–	–
Diabetes duration, yr	8.32 ± 6.91	–	–	7.74 ± 6.82	–	–
HbA1c, %	9.05 ± 2.51	–	–	9.13 ± 2.54	–	–
FPG, mM	7.86 ± 3.22	5.05 ± 0.47	<0.001	7.91 ± 3.18	5.06 ± 0.50	<0.001
BMI, kg/m^2^	24.7 ± 3.75	23.6 ± 3.37	<0.001	24.9 ± 3.93	23.7 ± 3.24	<0.001
Triglycerides, mM	1.87 ± 2.01	1.49 ± 0.96	0.002	2.04 ± 2.38	1.58 ± 1.02	<0.001
HDL cholesterol, mM	1.07 ± 0.30	1.41 ± 0.35	<0.001	1.10 ± 0.31	1.38 ± 0.34	<0.001
LDL cholesterol, mM	2.90 ± 1.58	2.81 ± 0.68	NS	2.94 ± 1.37	2.83 ± 0.67	NS
Systolic BP, mm Hg	143 ± 22.4	124 ± 16.6	<0.001	141 ± 22.9	126 ± 17.7	<0.001
Diastolic BP, mm Hg	80.6 ± 11.3	75 ± 10.5	<0.001	80.9 ± 11.7	76 ± 11.1	<0.001

Data are expressed as the number (%) or average ± SD. Abbreviations used: HbA1c, haemoglobinA1c; FPG, Fasting Plasma Glucose; BMI, body mass index; BP, blood pressure; NS, no significant difference compared between cases and controls.

**Table 2:** Stage 1 analysis showed the association of SNPs in *(s)ANK1* for T2D development and the levels of HbA1c, Hb and fasting glucose.

	Gene	Effect/other	T2D Development^a^	HbA1c(%)^b^	HbA1c (%) adjusted for glucose, sex, age^b^	Hb (g/L)^b^	Hb (g/L) adjusted for sex, age^b^	Fasting glucose (mmol/L)^b^	Fasting glucose (mmol/L) adjusted for sex, age^b^
rs4466386	*ANK1*	T/C	0.969 (0.789–1.191)	−0.045 (0.012)	−0.036 (0.014)	−0.10 (0.001)	−0.01 (0.002)	−0.008 (0.01)	0.002 (0.01)
			0.768	0.365	0.52	0.838	0.849	0.875	0.963
rs10090395	*ANK1*	C/T	1.002 (0.773–1.300)	0.054 (0.01)	0.042 (0.011)	0.038 (0.001)	0.031 (0.001)	0.027 (0.008)	0.015 (0.008)
			0.983	0.276	0.454	0.43	0.55	0.583	0.762
rs4737009	*ANK1*	A/G	1.034 (0.854–1.253)	−0.035 (0.013)	−0.056 (0.015)	0.051 (0.002)	0.041 (0.002)	0.018 (0.01)	0.007 (0.01)
			0.732	0.476	0.315	0.288	0.438	0.716	0.893
rs6474359	*ANK1*	C/T	1.010 (0.654–1.559)	−0.036 (0.007)	0.001 (0.007)	−0.61 (0.001)	−0.101 (0.001)	−0.031 (0.005)	−0.024 (0.005)
			0.963	0.464	0.987	0.213	0.054	0.528	0.623
rs508419	*(s)ANK1*	C/T	1.663 (1.247–2.218)	0.102 (0.009)	0.119 (0.01)	0.023 (0.001)	−0.021 (0.001)	−0.006 (0.007)	−0.018 (0.007)
			4.83 × 10^−4^	0.040	0.034	0.634	0.691	0.902	0.709
rs515071	*(s)ANK1*	C/T	1.583 (1.228–2.039)	0.104 (0.01)	0.115 (0.011)	0.049 (0.001)	0.026 (0.001)	−0.012 (0.007)	−0.026 (0.007)
			3.63 × 10^−4^	0.034	0.040	0.312	0.627	0.801	0.593
rs516946	*(s)ANK1*	C/T	1.750 (1.308–2.341)	0.106 (0.008)	0.121 (0.009)	0.032 (0.001)	−0.010 (0.001)	−0.004 (0.007)	−0.018 (0.007)
			1.41 × 10^−4^	0.032	0.029	0.513	0.856	0.939	0.718
rs4737000	*(s)ANK1*	C/A	0.972 (0.790–1.195)	0.072 (0.014)	0.085 (0.015)	−0.027 (0.002)	−0.041 (0.002)	−0.029 (0.01)	−0.039 (0.011)
			0.788	0.148	0.127	0.575	0.436	0.549	0.427

^a^Data were showed as OR (95% CI) (upper) and p value (lower).

^b^Data were showed as β (SE) (upper) and p value (lower).

**Table 3:** Stage 2 analysis showed the association of SNPs in (s)*ANK1* for T2D development and the levels of HbA1c, Hb and fasting glucose.

	T2D Development^a^	HbA1c (%) adjusted for glucose, sex, age^b^	Hb (g/L) adjusted for sex, age^b^	Fasting glucose (mmol/L) adjusted for sex, age^b^
SNP (Effect/other)				
rs508419 (C/T)	1.531 (1.215–1.930)2.85 × 10^−4^	0.069 (0.008)0.115	0.005 (0.001)0.898	−0.015 (0.005)0.696
rs515071 (C/T)	1.468 (1.199–1.798)1.86 × 10^−4^	0.049 (0.008)0.261	0.047 (0.001)0.263	0.008 (0.006)0.828
rs516946 (C/T)	1.495 (1.187–1.882)5.88 × 10^−4^	0.071 (0.007)0.106	0.000 (0.001)0.996	−0.005 (0.005)0.893
CCC	1.477 (1.203–1.814)1.87 × 10^−4^	1.292 (0.930 ~ 1.794)0.125	0.711 (0.491 ~ 1.029)0.069	0.817 (0.578–1.154)0.251
rs4737009 (A/G)	−	−0.031 (0.012)0.488	0.025 (0.001)0.473	0.026 (0.008)0.495
rs6474359 (C/T)	−	−0.001 (0.005)0.978	−0.059 (0.001)0.161	− 0.027 (0.004)0.481

^a^Data were showed as OR (95% CI) (upper) and p value (lower).

^b^Data were showed as β (SE) (upper) and p value (lower) after adjusted.
