# Supplementary material for: A novel type 2 diabetes risk allele increases the promoter activity of the muscle-specific small ankyrin 1 gene
Source: Sci Rep. 2016 Apr 28;6:25105. doi: 10.1038/srep25105 (PMC4848520; doi:10.1038/srep25105)
Supplement: Supplementary Information [file srep25105-s1.doc]

**A novel type 2 diabetes risk allele increases the promoter activity of the muscle-specific small ankyrin 1 gene**

Rengna Yan^1,2,7^,Shanshan Lai^1,5,6^, Yang Yang^1,3,6^, Hongfei Shi^1,4,6^, Zhenming Cai^1,6^, Vincenzo Sorrentino^8^, Hong Du^1,2,**^, Huimei Chen^1,6,*^

^1^School of Medicine, Nanjing University, Nanjing, 210093, China;^2^Department of Endocrinology, Jinling Hospital Affiliated to Nanjing University School of Medicine, Nanjing, 210002, China;^3^Department of Urology, Drum Tower Hospital Affiliated to Nanjing University School of Medicine, Nanjing, 210008, China;^4^Department of Orthopedics, Drum Tower Hospital Affiliated to Nanjing University School of Medicine, Nanjing, 210008, China;^5^MOE Key Laboratory of Model Animals for Disease Study, Model Animal Research Center and the School of Medicine, Nanjing University, National Resource Center for Mutant Mice, Nanjing 210093, China;^6^Jiangsu Key Laboratory of Molecular Medicine, Nanjing, 210002, China;^7^Department of Endocrinology, Nanjing First Hospital Affiliated to Nanjing Medical University, Nanjing, 210006, China;^8^Molecular Medicine Section, Department of Molecular and Developmental Medicine, University of Siena, Siena, 53100, Italy

*******Corresponding author:** Huimei Chen

The Department of Medical genetics, Nanjing University School of Medicine

Hankou Road 22, Nanjing 210093, China

Tel: +86-25-83686041, Fax: +86-25-83686451

Email: [chenhuimei@nju.edu.cn](mailto:chenhuimei@nju.edu.cn)

**** Co-corresponding author:** Hong Du

Jinling Hospital, Nanjing University School of Medicine, Nanjing, China

Zhongshan East Road 305, Nanjing, 210002, China

Tel: +86-13951791208, Fax: +86- 25-80860155

Email: [duhong5@126.com](mailto:duhong5@126.com)

**Supplementary material**

**Supplementary Figure S1.** **EMSA of C2C12 cell extracts specific bound to the rs508419T allele**


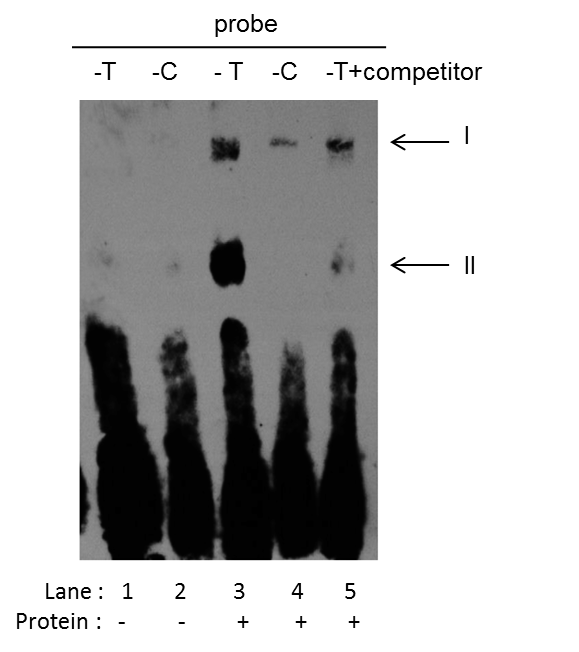


Lane 1 and lane 2, no extract; lane 3 and lane 4, without competitor; lane 5, rs508419T probe, with a 100-fold excess of rs508419T probe as competitor.

**Supplementary Figure S2.** **EMSA analysis with 3T3-L1 cell extracts and oligonucleotide probes containing C and T alleles of ANK1 P1promoter.**

**
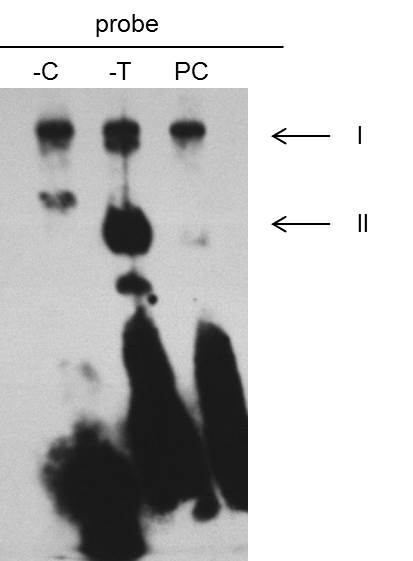
**

Except C2C12, sAnk1 was also found enriched in 3T3-L1 [^1^](#_ENREF_1), so EMSA analysis were produced in 3T3-L1 cell line. PC, positive control.

**Supplementary Figure S3.** **Genomic structure of *ANK1* and location of primers used to evaluate sAnk1 expression.**


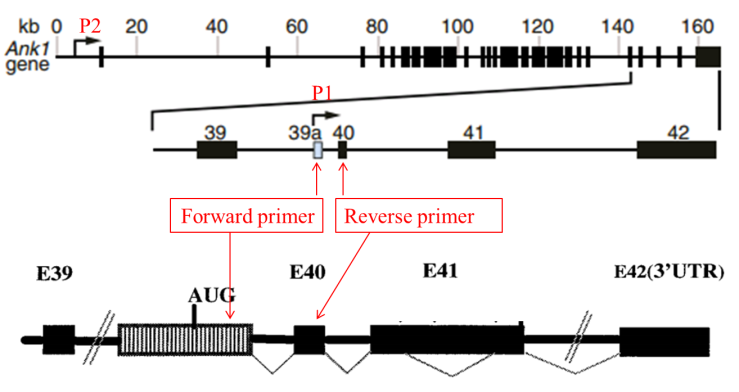


P1, Promoter 1; P2, Promoter 2

**Supplementary Figure S4. Correlation between sAnk1 and SERCA1 and between sAnk1 and GLUT4 levels in skeletal muscle of mice.**


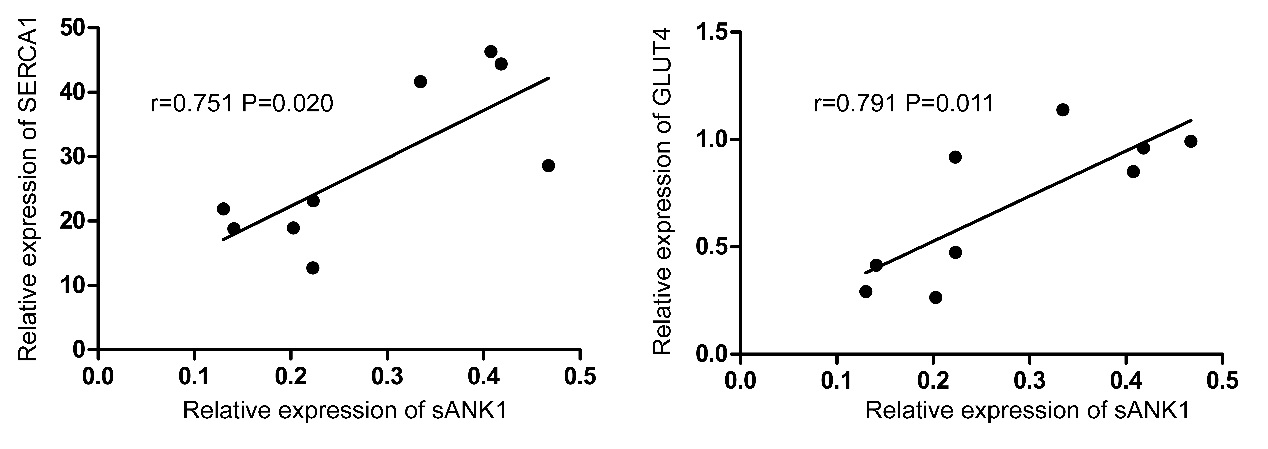


Skeletal muscle tissues were randomly collected from db/db (n=5) and db/m (n=5) mice. After the RNA extraction and cDNA synthesis, qPCR was used to evaluate the relative mRNA levels of sAnk1, SERCA and Glut4 compared to ARPPO with 2^-Δt^. Pearson correlation analysis showed that sAnk1 is highly correlated with SERCA1 (r= 0.751, p= 0.02) and GLUT4 (r= 0.791, p= 0.011) levels. The sequences of the primers used for quantitative PCR (qPCR) are shown in Supplementary Table S4.

**Supplementary Table S1. Candidate SNPs selected at the (s)ANK1 locus.**

| resSNP ID | Position | Roughly location | Variation | MAF^a^ | H-W^b^ | Studies |
| --- | --- | --- | --- | --- | --- | --- |
| rs4737000 | 41628884 | 3'-flanking in ANK1 and sANK1 | A/C | 0.427 | 0.656 | in this study |
| rs508419 | 41642148 | Intron 39 in ANK1; 5’- flanking in sANK1 | C/T | 0.171 | 0.391 | in this study |
| rs10090395 | 41786031 | 5’-flanking in ANK1 | T/C | 0.110 | 0.233 | in this study |
| rs4466386 | 41799010 | 5’-flanking in sANK1 | T/C | 0.329 | 0.101 | in this study |
| rs516946 | 41638405 | Intron 41 in ANK1; intron 2 in sANK1 | C/T | 0.110 | 0.358 |  |
| rs515071 | 41638619 | Intron 40 in ANK1; intron1 in sANK1 | C/T | 0.195 | 0.305 | [^1^](#_ENREF_1) |
| rs6474359 | 41668351 | Intron 31 in ANK1 | T/C | 0.027 | 0.366 | [^4^](#_ENREF_4) |
| rs4737009 | 41749562 | 5’-flanking in ANK1 | A/G | 0.463 | 0.85 | [^4^](#_ENREF_4) |

Abbreviation: MAF, Minor allele frequency, H-W, Hardy-Weinberg equilibrium.

a. the MAFs were listed based on public data from pubmed.

b. H-W indexes were calculated based on the data in controls of Stage 1.

**Supplementary Table S2. Sequences of PCR Primers used for genotyping.**

| SNP (Alleles) | Genotyping | Primer sequence | Annealing temperature (℃) | Product length (bp) |
| --- | --- | --- | --- | --- |
| rs4737000  (A/C) | PCR-RFLP | F: 5'-CAGATGGACTTCTGCTCCTC-3'  R: 5'-ATAACCTAAATCCCTGTGC-3' | 54 | 257 |
| rs515071  (C/T) | PCR-RFLP | F: 5'-GATCTGCACGTCAGCCTC-3'  R: 5'-AATATCGACCTCCAGCTCACT-3' | 60 | 212 |
| rs4463866  (T/C) | HRM | F: 5'-ATACACGTCCAAGCTCAC-3'  R: 5'-ATTGTACTCTGCCAAACG-3' | 53 | 115 |
| rs10090395  (T/C) | HRM | F: 5'-AAACAAACACTACATCTACACC-3'  R: 5'-CATGGTTTTGAGTATCACACAC-3' | 53 | 150 |
| rs508419  ( C/T) | HRM | F: 5'-TTGGATCACCGCAGTAGCA-3'  R: 5'-ACACCCGCTGCTGCCTAA-3' | 63 | 193 |
| rs516946  (C/T) | TaqMan Genotype Assay | Assay ID: C__10008819_10 | | |
| rs6474359  (T/C) | TaqMan Genotype Assa | Assay ID: C__31336804_10 | | |
| rs4737009  (G/A) | TaqMan Genotype Assa | Assay ID: C__32337793_10 | | |

**Supplementary Table S3. Demographics information of the muscles from the volunteers.**

|  |  | rs508419 | | |  |
| --- | --- | --- | --- | --- | --- |
|  | Total | CC | CT | TT | *P* |
| Volunteers, n (%) | 24 | 17 | 5 | 4 |  |
| Male, n (%) | 10 (41.7) | 6 (35.3) | 2 (40) | 2 (50) | NS |
| Age, yr (±SD) | 47.3 (21.5) | 43.9 (23.2) | 57.6 (4.6) | 59.5 (6.4) | NS |

NS, no significance compared between different genotypes.

**Supplementary Table S4. Sequences of primers used for real-time PCR.**

| PCR primers | **Sequences** |
| --- | --- |
| hsAnk1-F | 5’-TCAGTGACGACGAGGAGACC-3’ |
| hsAnk1-R | 5’-TCCTCTGTCACCTGCTCCC-3’ |
| msAnk1-F | 5’-CTGGTGCTGTTAGGCTTCTTC-3’ |
| msAnk1-R | 5’-GTTCCTGGTGGATGTGCTTC-3’ |
| mSERCA1-F | 5’-TGTTTGTCCTATTTCGGGGTG-3’ |
| mSERCA1-R | 5’-AATCCGCACAAGCAGGTCTTC-3’ |
| mGLUT4-F | 5’-TTCCTTCTATTTGCCGTCCTC-3’ |
| mGLUT4-R | 5’-TGGCCCTAAGTATTCAAGTTCTG-3’ |
| hARPPPO-F | 5’-GAAACTGCTGCCTCATATCCG-3’ |
| hARPPPO-R | 5’-GCTGGCACAGTGACTTCACATG-3’ |
| mARPPPO-F | 5’-GAAACTGCTGCCTCACATCCG-3’ |
| mARPPPO-R | 5’-GCTGGCACAGTGACCTCACACG-3’ |

**Supplementary Table S5. Primer sequences for plasmids construction.**

| Plasmids | Primer sequences | Restriction sites |
| --- | --- | --- |
| Promoter 1 | Forward: 5’-GGGGTACCgttcctgctcagtgggcgt-3’  Reverse: 5’-GGAGATCTggcaggacaccgaatgg-3’ | KpnI  BglII |
| Promoter 2 | Forward: 5’-GGGGTACCgaactctttctagttcccatgg-3’  Reverse: 5’-GGAGATCTggcaggacaccgaatgg-3’ | KpnI  BglII |

**Supplementary Table S6. Sequences of EMSA probe.**

| EMSA probe | Sequences |
| --- | --- |
| EMSA probe-C | 5’-gcaggataggCgagaggccgag-Biotin-3’  3’-Biotin-cgtcctatccgctctccggctc-5’ |
| EMSA probe-T | 5’-gcaggataggTgagaggccgag-Biotin-3’  3’-Biotin-cgtcctatccactctccggctc-5’ |

**Supplementary Method**

**Specific genotyping for either SNPs.**

Three SNPs (rs516946, rs4737009, and rs6474359) were genotyped using TaqMan Genotype assays (Applied Biosystems, Inc.), according to the manufacturer’s protocol using the Viia 7 Real-Time PCR system (Applied Biosystems). For quality control, no template controls (NTCs) were simultaneously detected in each 384-well plate.

Genotyping for the rs508419, rs4466386, and rs10095395 SNPs was performed by HRM analysis. PCR amplification was performed in a 10-uL volume containing 25 ng genomic DNA, 0.2 pmol each primer, 1 uL 10 × Taq buffer with (NH_4_)_2_ SO_4_, 1 uL 25 mM MgCl_2_, 0.8 uL 2.5 mM dNTPs, 1 uL 10 × LC Green PLUS (Idaho Technology, Salt Lake City, UT, USA), 0.4 U Taq DNA Polymerase (Fermentas, St. Leon Rot, Germany), and 0.4 uL DMSO. The reaction mixtures were incubated at 95°C for 5 min and then subjected to 40 cycles of 95°C for 30 s, 54–63°C (Supplementary Table S2) for 30 s, and 72°C for 30 s, followed by 72°C for 7 min using a PTC-200 thermal cycler (Bio-Rad Laboratories, Berkeley, CA, USA). After PCR was completed, genotyping of the polymorphisms was performed by HRM analysis, as previously described [^5^](#_ENREF_5). For confirmation of the results, 10% of the samples from each group detected by HRM were randomly selected and subjected to DNA sequencing.

For rs515071 and rs4737000, genotyping was performed by PCR-RFLP analysis. After digestion with an appropriate restriction enzyme (*Xsp*I or *Hinf*I for rs515071 and rs4737000, respectively; Takara, Shiga, Japan), the PCR products were separated on a 3% agarose gel to assess the fragment polymorphisms in the digested *ANK1* amplicon. To validate the genotyping results, duplicated genotyping was performed in 10% of the samples.

**mRNA expression analysis in mice.**

Skeletal muscle tissues were randomly collected from db/db (n=5) and db/m (n=5) mice. Muscle tissues were obtained at biopsy and immediately frozen in liquid nitrogen and stored at −70°C until further analysis. Total RNA was extracted from the skeletal muscle samples using the TRIzol method (Takara), and cDNA synthesis was performed using the PrimeScript RT Reagent Kit (Takara). Real-time PCR was used to quantify mRNA expression levels of sAnk1 using primers that specifically amplify this isoform. Relative cDNA expression of sAnk1, SERCA and Glut4 detected by qPCR were calculated using the 2^-ΔCT^ method (AB7300 real-time PCR system, Applied Biosystems). The sequences of the primers used for quantitative PCR (qPCR) are shown in Supplementary Table S4.

**References**

1. Imamura, M. et al. A single-nucleotide polymorphism in ANK1 is associated with susceptibility to type 2 diabetes in Japanese populations. *Human Molecular Genetics* **21**, 3042-3049 (2012).

2. Morris, A.P. et al. Large-scale association analysis provides insights into the genetic architecture and pathophysiology of type 2 diabetes. *Nat Genet* **44**, 981-90 (2012).

3. Harder, M.N. et al. Type 2 Diabetes Risk Alleles Near BCAR1 and in ANK1 Associate With Decreased  -Cell Function Whereas Risk Alleles Near ANKRD55 and GRB14 Associate With Decreased Insulin Sensitivity in the Danish Inter99 Cohort. *Journal of Clinical Endocrinology & Metabolism* **98**, E801-E806 (2013).

4. Soranzo, N. et al. Common Variants at 10 Genomic Loci Influence Hemoglobin A1C Levels via Glycemic and Nonglycemic Pathways. *Diabetes* **59**, 3229-3239 (2010).

5. Sun, C. et al. Functional polymorphism of hOGG1 gene is associated with type 2 diabetes mellitus in Chinese population. *Mol Cell Endocrinol* **325**, 128-34 (2010).
